# Single-cell RNA sequencing of submandibular gland reveals collagen type XV-positive fibroblasts as a disease-characterizing cell population of IgG4-related disease

**DOI:** 10.1186/s13075-024-03289-7

**Published:** 2024-02-20

**Authors:** Shigeru Tanaka, Takuya Yamamoto, Arifumi Iwata, Masahiro Kiuchi, Kota Kokubo, Tomohisa Iinuma, Takahiro Sugiyama, Toyoyuki Hanazawa, Kiyoshi Hirahara, Kei Ikeda, Hiroshi Nakajima

**Affiliations:** 1 Department of Allergy and Clinical Immunology, Graduate School of Medicine, Chiba University, 1-8-1 Inohana, Chiba, 260–8670 Japan; 2 Department of Immunology, Graduate School of Medicine, Chiba University, Chiba, Japan; 3 Department of Otorhinolaryngology/Head & Neck Surgery, Graduate School of Medicine, Chiba University, Chiba, Japan; 4 Department of Rheumatology, Dokkyo Medical University, 880 Kitakobayashi, Shimotsuga, Tochigi, Mibu, 321 − 0293 Japan

**Keywords:** IgG4-related disease, Fibroblasts, Collagen type XV, Single-cell RNA sequence

## Abstract

**Objectives:**

IgG4-related disease (IgG4-RD) is a systemic autoimmune disease with an unknown etiology, affecting single/multiple organ(s). Pathological findings include the infiltration of IgG4-producing plasma cells, obliterative phlebitis, and storiform fibrosis. Although immunological studies have shed light on the dysregulation of lymphocytes in IgG4-RD pathogenesis, the role of non-immune cells remains unclear. This study aimed to investigate the demographics and characteristics of non-immune cells in IgG4-RD and explore potential biomarkers derived from non-immune cells in the sera.

**Methods:**

We conducted single-cell RNA sequence (scRNA-seq) on non-immune cells isolated from submandibular glands of IgG4-RD patients. We focused on fibroblasts expressing collagen type XV and confirmed the presence of those fibroblasts using immunohistochemistry. Additionally, we measured the levels of collagen type XV in the sera of IgG4-RD patients.

**Results:**

The scRNA-seq analysis revealed several distinct clusters consisting of fibroblasts, endothelial cells, ductal cells, and muscle cells. Differential gene expression analysis showed upregulation of *COL15A1* in IgG4-RD fibroblasts compared to control subjects. Notably, *COL15A1*-positive fibroblasts exhibited a distinct transcriptome compared to *COL15A1*-negative counterparts. Immunohistochemical analysis confirmed a significant presence of collagen type XV-positive fibroblasts in IgG4-RD patients. Furthermore, immune-suppressive therapy in active IgG4-RD patients resulted in decreased serum levels of collagen type XV.

**Conclusions:**

Our findings suggest that collagen type XV-producing fibroblasts may represent a disease-characterizing non-immune cell population in IgG4-RD and hold potential as a disease-monitoring marker.

**Supplementary Information:**

The online version contains supplementary material available at 10.1186/s13075-024-03289-7.

## Introduction

IgG4-related disease (IgG4-RD) is an autoimmune disease characterized by the infiltration of IgG4-positive plasma cells, obliterative phlebitis, and storiform fibrosis [1]. Although the etiology of this disease remains largely unknown, recent studies have shed light on the significant roles of T- and B-lymphocytes [2–5]. While there have been notable advances in understanding the involvement of immune cells in the pathology of IgG4-RD, limited insight presently exists regarding the implications of non-immune cells on IgG4-RD pathogenesis, as well as their distinctive characteristics [6]. Notably, unlike other fibrotic diseases such as systemic sclerosis and idiopathic pulmonary fibrosis, the fibrotic changes observed in IgG4-RD are generally reversible, indicating the existence of distinct profibrotic machinery [7].

One promising approach for elucidating the pathogenic cell subsets in various diseases is single-cell RNA sequencing (scRNA-seq). This relatively new technique allows for the exploration of novel cell subsets in the affected organs. In the context of IgG4-RD, scRNA-seq has been applied to analyze peripheral blood mononuclear cells, revealing the heterogeneity of monocytes, T-lymphocytes, and B-lymphocytes [8]. However, only a few reports have utilized scRNA-seq to analyze non-immune cells in the affected tissues [9], which could potentially provide crucial insights into the pathogenesis of IgG4-RD.

In this study, we conducted scRNA-seq analysis of submandibular gland tissues from patients with IgG4-RD. Our findings unveil the presence of a disease-related cell subset, namely collagen type XV-expressing fibroblasts, which may have distinct functions. Furthermore, we demonstrate that serum levels of collagen type XV could serve as a monitoring tool for IgG4-RD patients.

## Methods

### Patients

IgG4-RD patients who fulfilled the 2019 American College of Rheumatology/European League Against Rheumatism Classification Criteria for IgG4-Related Disease [10] were enrolled in this study. As controls for scRNA-seq analyses, patients with oropharyngeal cancer who underwent neck dissection surgery were also included. Healthy volunteers were recruited as controls for serum collagen type XV assessment. Patients with active disease were defined as those who have received systemic immunosuppressive therapy due to severe glandular symptoms leading to a decrease in vision or exhibit visceral lesions. Written informed consent was obtained from all participants in accordance with the Declaration of Helsinki. Tables 1 and 2 provide an overview of the demographic characteristics of the participants. Supplementary Table S1 provides clinical manifestation of patients and control subject.

**Table 1:** Clinical features of IgG4-RD patients and control subject analyzed using scRNA-seq and IHC

	IgG4-RD (*N* = 4)	Control (*N* = 3)
Age, median (IQR), years	62 (55.75–74)	60 (57.5–61)
Sex, female (%)	0	0
number of involved organs, median (IQR)	2 (1.75–2)	N.D.
*Serum IgG4, median (IQR), mg/dL	1333 (925–1700)	N.D.
**Serum IgG, median (IQR), mg/dL	2519 (2390–3058)	N.D.
IgG4 responder index, median (IQR)	8 (8–8.75)	N.D.

N.D.: not determined, scRNA-seq: single-cell RNA sequencing, IHC: immunohistochemistry

*Serum IgG4 normal range: 11–121 mg/dL

**Serum IgG normal range: 870–1700 mg/dL

**Table 2:** Clinical features of IgG4-RD patients and control participants subjected to serum collagen type XV measurement

	HC (*N* = 5)	IgG4-RD, inactive (*N* = 5)	IgG4-RD, active (*N* = 10)
Age, median (IQR), years	69 (59–73)	69 (66–69)	65 (64–69.25)
Sex, female (%)	40	20	20
number of involved organs, median (IQR)	N.D.	1 (1–2)	2 (1.25–2)
*Serum IgG4, median (IQR), mg/dL	N.D.	397 (220–480)	1073 (437.5–1240)
*Serum IgG4 3 months after treatment, median (IQR), mg/dL	N.D.	N.D.	173 (93.5–252.75)
IgG4-RD responder index before treatment, median (IQR)	N.D.	0 (0–1)	11.5 (8.5–13.5)
IgG4-RD responder index 3 months after treatment, median (IQR)	N.D.	N.D.	1 (0.25–1.75)

HC: healthy control, IgG4-RD: IgG4 related disease, N.D.: not determined

*Serum IgG4 normal range: 11–121 mg/dL

### Submandibular gland preparation

Human submandibular gland samples were placed in a C tube (Miltenyi Biotec, North Rhine-Westphalia, German) containing digestion buffer (Multi Tissue Dissociation Kit 1, Miltenyi Biotec), and tissues were dissociated by a gentleMACS™ octo dissociator with heaters (Miltenyi Biotec). Submandibular gland cells in cell suspension were separated using Percoll solution (GE Healthcare, Chicago, IL). Subsequently, cells were stained with anti-human CD45 PE (BD biosciences, Franklin Lakes, NJ) antibodies. Non-immune cells (CD45-) were purified from stained cells using a BD Aria III (BD).

### scRNA-seq

Cells from submandibular glands were encapsulated into droplets, and libraries were prepared using Chromium Single Cell 3.1’ Reagent Kits v3 according to the manufacturer’s protocol (10X Genomics, Pleasanton, CA). The generated scRNA-seq libraries were sequenced with a NovaSeq 6000 (Illumina, San Diego, CA). Sequence reads from all samples were processed and aggregated using a Cell Ranger (version 6.0) (10X Genomics). Aggregated data were further analyzed by Seurat [11]. Specifically, we first removed sex chromosome-related genes from the analyses because of the imbalance for the participant’s sex. We applied SoupX to remove ambient RNA from the data. After this procedure, we log-normalized the expression matrix and regressed the data against the total number of unique molecular identifiers (UMIs) detected per cell. For each sample, poor-quality cells were filtered out using the following criteria: (1) the percentage of UMIs derived from mitochondrial genes above 10%, and (2) the number of detected genes below 700. Cells expressing hematopoietic markers (*PTPRC, CD3E, TRAC, TRDC, NKG7, GZMA, CD79A, IGHG1, IGHG2, IGHG3, IGHG4, IGHM, IGHE, MS4A1, PF4, FCGR3A, MS4A7, FCER3A*, and *ITGAX*) were also excluded from the analysis. Subsequently, the data were subjected to principal component analysis (PCA), and we used PCA dimensions 1–15 to find clusters on a Uniform Manifold Approximation and Projection (UMAP) analysis. The marker genes of each cluster and differentially expressed genes between samples were identified by FindMarkers in Seurat. Gene set enrichment analysis (GSEA) was performed on differentially expressed genes using clusterProfiler (v4.8.1) [12].

### Immunohistochemistry

Collagen type I expression was determined as an indicator for fibrosis [13], and collagen type XV was identified as a disease-specific molecule. Fresh frozen submandibular gland samples were subjected to immunohistochemical analysis. The slides were thawed and washed in PBS, followed by blocking with 5% goat serum at room temperature for 1 h in a humid chamber. Subsequently, the slides were treated with the following primary antibodies: anti-collagen I alpha 1 antibody (1:1000, COL-1, Novus Biological, Centennial, CO) and anti-COL15A1 antibody (1:200, polyclonal, Merck, Rahway, NJ). The slides were then incubated overnight at 4 ºC. Following overnight incubation, the slides were washed and incubated with the appropriate secondary antibodies at room temperature for 1 h in the dark. The secondary antibodies used were as follows: Alpaca anti-mouse IgG1 antibody conjugated with Alexa Fluor 488 (1:250, Thermo Fisher Scientific) and goat anti-rabbit IgG antibody conjugated with Alexa Fluor 647 (1:200, Thermo Fisher Scientific). Nuclear DNA was counterstained with DAPI and visualized using an EVOS M7000 (Thermo Fisher Scientific). Collagen type XV-positive cells were defined as being absent in the basement membrane region, having an isolated nucleus, and exhibiting collagen type XV staining in the plasma membrane/cytoplasm. The number of collagen type XV-positive cells between the control and IgG4-RD groups was compared by analyzing five randomly selected fields of view captured using a 20x lens.

### Enzyme-linked immunosorbent assay (ELISA)

Serum collagen type XV levels were determined by using a Human Collagen Type XV ELISA kit (Elabscience, cat# E-EL-H0772, Houston, TX) according to the manufacturer’s protocol.

### Statistical analyses

Statistical analyses were performed using GraphPad Prism 10 (GraphPad, San Diego, CA). The number of collagen type XV-positive cell were analyzed using an unpaired t-test. Serum levels of type XV collagen were analyzed using One-way ANOVA test followed by Kruskal-Wallis test and paired t-test. P values less than 0.05 were considered statistically significant.

### Data availability

RNA sequence data are submitted to the Gene Expression Omnibus (GEO). The data is available under the accession number GSE242778.

## Results

### *COL15A1* expression is upregulated in fibroblasts in IgG4-RD

**Fig. 1:**
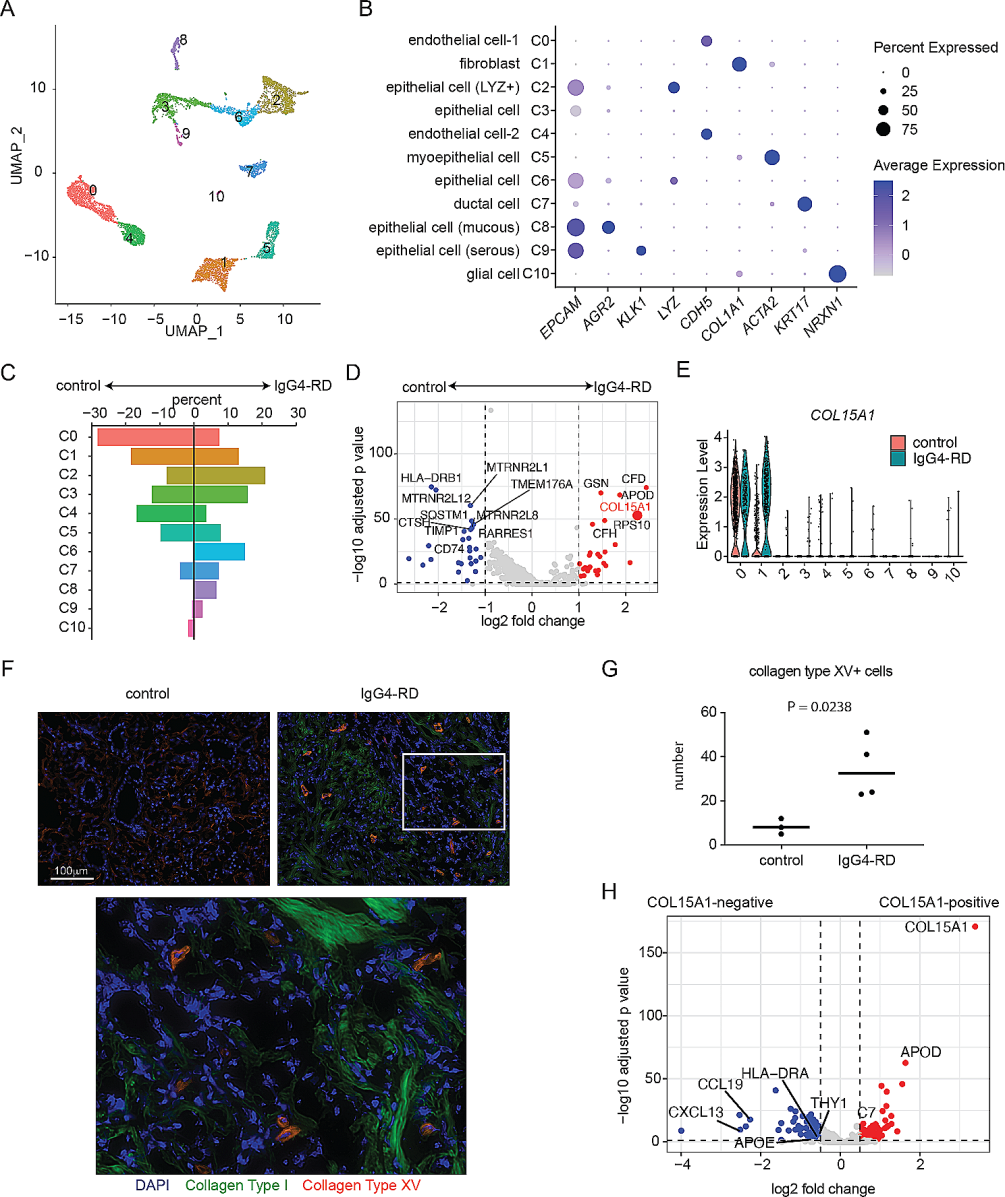
Disease-related fibroblasts express collagen type XV in IgG4-RD submandibular gland. **(A-E)** Single-cell RNA sequencing analyses performed on non-hematopoietic cells from the submandibular glands of patients with IgG4-RD and controls. *N* = 2 for each group. **(A)** The UMAP plot shows that the presence of 11 clusters within the non-hematopoietic cells of the submandibular gland. **(B)** The characteristic gene expression patterns within each cluster, accompanied by the corresponding predicted cluster names. **(C)** The bar graph represents the proportions of each cluster. **(D)** The volcano plot shows the differentially expressed genes in fibroblasts (C1). **(E)** The expression of *COL15A1* in each cluster. **(F-G)** Immunohistochemistry analysis of collagen type XV fibroblast in submandibular gland. **(F)** Representative images of the submandibular gland from control subjects and patients with IgG4-RD. For the IgG4-RD sample, a high magnification image is also included. **(G)** Cumulative data on the number of collagen type XV-positive cells is shown. **(H)** The differentially expressed genes between *COL15A1*-positive and *COL15A1*-negative cells

The involvement of non-immune cell components in the pathogenesis of IgG4-RD remains a subject of ongoing debate and uncertainty. In order to gain insights into the specific cell types that have the potential to promote the disease, we performed scRNA-seq analysis on non-hematopoietic cells in the submandibular gland tissue of IgG4-RD patients. As a control group, we also analyzed the submandibular gland tissue from head and neck cancer patients who underwent lymph node dissection. Our analysis revealed the presence of eleven cell clusters, including fibroblasts, endothelial cells, epithelial cells, ductal cells, muscle cells, and glial cells (Fig. 1A and B). Fibroblasts (C1) were found to be abundant in both IgG4-RD and control samples, with no significant differences in their proportions (Fig. 1C). Unexpectedly, the percentage of endothelial cells (C0 and C4) was decreased in IgG4-RD, whereas the percentage of epithelial cells (C2, C3, C6, C8, and C9) was increased (Fig. 1C). Myoepithelial cells appeared to be unchanged between IgG4-RD and control samples (Fig. 1C).

Subsequently, we performed a focused differential gene expression analysis, centering particularly on the fibroblast cluster. This choice is rooted in the pivotal role played by fibroblasts as the primary cellular component responsible for tissue fibrosis, a hallmark feature of IgG4-RD. We observed 66 upregulated genes and 397 downregulated genes in IgG4-RD fibroblasts compared to controls (Fig. 1D). Among these genes, we specifically examined *COL15A1*, which encodes collagen type XV. Collagen type XV is known to anchor cells to the basement membrane and is mainly present around lumens [14]. In line with this understanding, we found the expression of *COL15A1* in endothelial cells (Fig. 1E). In control samples, only a few fibroblasts expressed *COL15A1*; however, in IgG4-RD samples, a substantial number of fibroblasts showed *COL15A1* expression (Fig. 1E). We also examined the expression of other collagen family members, such as *COL1A1*, but no significant differences were observed between IgG4-RD and control samples (Supplementary Figure S1). These findings suggest that the expression of *COL15A1* may be a disease-related characteristic of fibroblasts in IgG4-RD.

### Collagen type XV-positive fibroblasts are increased in IgG4-RD submandibular gland

Next, we investigated whether fibroblasts in the submandibular gland of IgG4-RD patients express collagen type XV protein and whether there is deposition of collagen type XV in the fibrotic tissues. To address this, we performed an immunohistochemical (IHC) assay. In this analysis, we included 4 IgG4-RD patients and 3 control participants. Collagen type XV exhibited faint staining as the basement membrane of the glandular tubular structure in the control samples (Fig. 1F). Only a small number of spindle-shaped cells expressed collagen type XV. In contrast, the glandular duct structures in the IgG4-RD samples were severely damaged, and the remaining glandular duct structures lacked lining by collagen type XV (Fig. 1F). The tissue showed a significant deposition of collagen type I, indicating a high level of fibrosis. Interestingly, the massive deposition of collagen type XV was not detected in fibrotic areas where collagen I was present. Instead, a considerable number of spindle-shaped and round cells exhibiting strong collagen type XV expression were observed (Fig. 1F and G). The collagen type XV-expressing cells were distributed ubiquitously (data not shown). The morphology of these cells suggested that the majority of them were fibroblasts. Collagen type XV-positive fibroblasts were found in regions that did not necessarily overlap with the fibrotic areas, making it challenging to consider them as the primary producers of collagen type I. These findings suggest that collagen type XV is not a major component of the extracellular matrix deposited in the fibrotic areas of IgG4-RD but rather could serve as a marker for disease-characterizing fibroblasts.

Based on our hypothesis that collagen type XV is a marker of disease-characterizing fibroblasts in IgG4-RD, we compared the gene expressions between *COL15A1*-positive and *COL15A1*-negative fibroblasts using scRNA-seq data. GO term enrichment analysis revealed that *COL15A1*-positive fibroblasts exhibited higher expression of morphology-related genes, whereas the expression of immune-related genes was decreased (Supplementary Figure S2). Interestingly, we observed an upregulation of *APOD* in *COL15A1*-positive fibroblasts (Fig. 1H). *APOD*, apolipoprotein D, is known as a senescent marker in aging skin [15], and it is also recognized as a characteristic gene expressed in peri-epithelial fibroblasts in the prostate [16]. These data suggest that collagen type XV-positive fibroblasts may demonstrate distinct functions compared to their collagen type XV-negative counterparts.

### The levels of serum collagen type XV decrease following systemic immune-suppressive therapy in treatment-naïve IgG4-RD patients

**Fig. 2:**
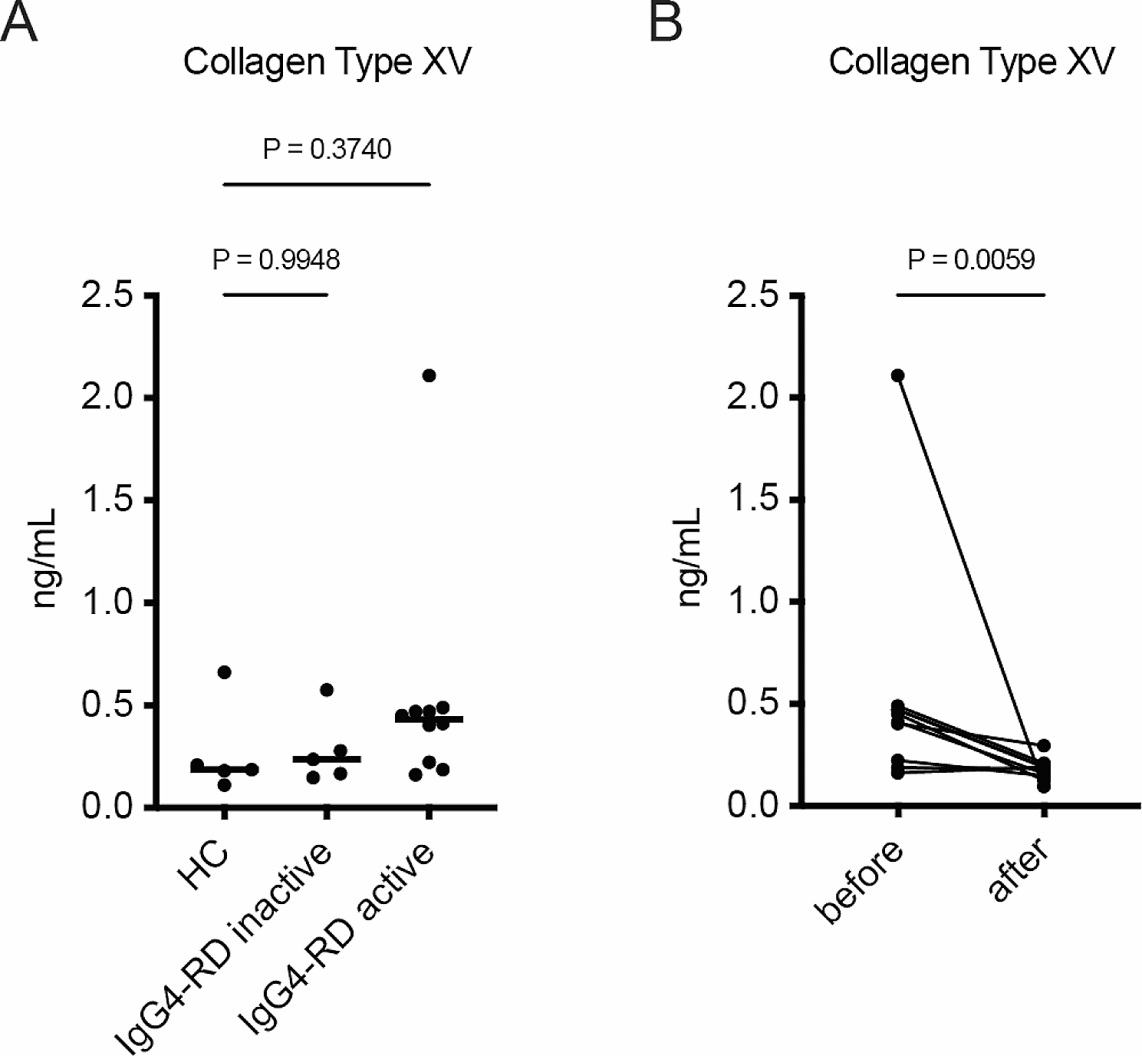
Serum collagen type XV levels are decreased after immune-suppressive therapy in patients with IgG4-RD. **(A)** The serum collagen type XV levels in healthy individuals (*N* = 5) and patients with IgG4-RD (*N* = 5 for inactive and *N* = 10 for active disease) are displayed. **(B)** The changes in serum collagen type XV levels before and after immune suppressive treatment in IgG4-RD patients are presented

Finally, we have determined the potential of collagen type XV as a biomarker for monitoring the disease activity. In this analysis, we employed serum collagen type XV levels due to the challenges associated with obtaining repeated tissue specimens. There were no apparent differences in serum collagen type XV levels among healthy, inactive patients, and active patients, indicating a lack of diagnostic power, possibly due to the small sample size (Fig. 2A). Among the patients with active disease, the values of collagen type XV were low in three individuals. There was no consistent trend observed in the backgrounds of these three patients (data not shown). Notably, a significant reduction in serum collagen type XV was observed following immune-suppressive therapy (Fig. 2B). In patients with low baseline collagen type XV values, there was no clear decrease observed after treatment. While it is challenging to conclude that serum collagen type XV concentration is useful for monitoring disease activity at the present stage based on these results, there is a potential indication of some clinical significance in patients with elevated collagen type XV levels at baseline.

## Discussion

Fibroblasts are essential cells involved in a range of biological processes, with their prominent role being the production of extracellular matrix. In this study, we identified a specific subset of fibroblast in IgG4-RD patients characterized by the expression of collagen type XV. Notably, collagen type XV-positive fibroblasts exhibited distinctive gene expression patterns. This discovery underscores the significance of comprehensively examining the unique properties and functions of collagen type XV-positive fibroblasts within the context of IgG4-RD.

Genome-wide association study (GWAS) revealed that *HLA-DRB1* is a disease susceptibility gene for IgG4-RD [17]. This observation indicates an interaction between CD4-positive T cells and antigen-presenting cells in the pathogenesis of IgG4-RD. Additionally, successful treatment with rituximab, aimed at removing B cells, indicates that the antibodies or B cells themselves are pathogenic [4, 18]. These studies suggest potential abnormalities in the acquired immune system and the presence of autoantigens in IgG4-RD. Interestingly, several reports have documented the deposition of IgG4 on the basement membrane of the pancreatic and bile duct [19, 20], indicating an immune response specifically targeted against a component of the basement membrane. As mentioned earlier, collagen type XV is mainly expressed on the basement membrane. Notably, collagen type XV expression in fibroblasts found in tissues from patients with IgG4-RD is significantly higher than in the basement membrane in healthy subjects. If collagen type XV is targeted by autoantibodies, it is possible that collagen type XV-positive fibroblasts are implicated in the pathogenesis of the disease through the production of autoantigens. In this context, the quantification of antibodies to collagen type XV in the bloodstream would be of significant interest.

Collagen type XV is known to be associated with pro-inflammatory responses. In a murine kidney injury model, collagen type XV and type XVIII mutant mice exhibit an attenuated influx of leukocytes in the ischemic kidney, suggesting that these collagens are crucial for the migration of leukocytes into the injured tissues [21]. It has also been shown that collagen type XV – integrin b1 interaction induces focal adhesion kinase (FAK) activation [22], resulting in endoplasmic reticulum (ER) stress-mediated inflammatory macrophage polarization [23]. Therefore, it is plausible that collagen type XV could promote tissue inflammation through the recruitment and activation of immune cells. To explore this further, it would be interesting to analyze immune cells infiltrated in the submandibular gland in IgG4-RD patients.

*APOD*, which we have identified as a characteristic gene of *COL15A1*-positive fibroblasts, is well known to be a component of high-density lipoprotein (HDL) [24]. The expression of ApoD in skin fibroblasts has been reported to be linked with cellular aging [15]. The process of skin tissue aging is believed to be influenced, in part, by an imbalance between extracellular matrix production and degradation [25]. Furthermore, tissue fibrosis is also characterized by abnormal matrix deposition and reduced degradation [26]. Considering this, the elevated expression of ApoD in collagen type XV-positive fibroblasts may be associated with fibrosis, as it may disrupt the extracellular matrix homeostasis within the tissue.

Interestingly, fibroblasts positive for *COL15A1* exhibited lower expression of immune-related genes such as *HLA-DRA*, *THY1*, and *CXCR13* (Fig. 1H). Previous studies have demonstrated that certain fibroblasts express MHC class II and possess the capability of antigen presentation [27–29]. Additionally, we observed a reduction in the expression of *CXCL13* in *COL15A1*-positive fibroblasts. It is worth noting that CXCL13-producing fibroblasts have been recognized as the crucial cells responsible for the formation of lymph follicles [30]. These findings suggest that fibroblasts expressing collagen type XV may not have a direct impact on the formation of lymphocyte aggregates observed in patients with IgG4-RD. It is evident that multiple phenotypes of fibroblasts exist, including those associated with tissue fibrosis pathology and those contributing to the immune response. Considering the absence of prominent features observed in the pathway analysis (Supplementary Fig. 2), it is possible that collagen type XV-expressing fibroblasts may not constitute an independent fibroblast subset but rather represent a condition associated with IgG4-RD. Further investigation is necessary for precise fibroblast classification and their functional analysis in IgG4-RD.

This study has several limitations. Firstly, the sample size of the enrolled participants was small. While IgG4-RD is considered a relatively rare disease, larger cohort studies are necessary to validate the presence and functional significance of collagen type XV-positive fibroblasts in IgG4-RD. Increasing the sample size would enhance the statistical power and reliability of the findings. Secondly, we focused exclusively on the analysis of non-hematopoietic cells and did not perform scRNA-seq for hematopoietic cells. Considering the emerging importance of cell-cell communication in disease pathology, it would be valuable to conduct scRNA-seq analysis for both hematopoietic and non-hematopoietic cells in the affected organs. Such an approach could provide comprehensive insights into the complex interactions between different cell types and potentially reveal additional factors contributing to the pathogenesis of IgG4-RD. Thirdly, there are some differences in the phenotype of submandibular fibroblasts recently reported, which were also characterized using scRNA-seq [9]. This discrepancy could potentially arise from the heterogeneity of IgG4-RD pathology and variations in the backgrounds of the control patients employed. Additional insights are anticipated to emerge as more tissue scRNA-seq data are accumulated from individuals affected by IgG4-RD.

Although many questions remain, this study offers valuable insights into the roles of non-hematopoietic cells in IgG4-RD. The identification of collagen type XV-positive fibroblasts as a specific fibroblast population associated with IgG4-RD opens up new avenues for potential therapeutic targeting in patients with IgG4-RD.

## Conclusions

Collagen type XV-producing fibroblasts may represent a disease-characterizing non-immune cell population in IgG4-RD.

### Electronic supplementary material

Below is the link to the electronic supplementary material.

Supplementary Material 1

## Data Availability

RNA sequence data are submitted to the Gene Expression Omnibus (GEO). The data is available under the accession number GSE242778.

## Abbreviations

IgG4-RD: IgG4-related disease
scRNA-seq: single-cell ribonucleotide acid sequence
ELISA: enzyme-linked immunosorbent assay
IHC: immunohistochemistry
GWAS: genome-wide association study
FAK: focal adhesion kinase
ER: endoplasmic reticulum
HDL: high-density lipoprotein
PCA: principal component analysis
UMAP: Uniform Manifold Approximation and Projection
