# Supplementary material for: Single-cell RNA sequencing of submandibular gland reveals collagen type XV-positive fibroblasts as a disease-characterizing cell population of IgG4-related disease
Source: Arthritis Res Ther. 2024 Feb 20;26:55. doi: 10.1186/s13075-024-03289-7 (PMC10877852; doi:10.1186/s13075-024-03289-7)
Supplement: Supplementary file 1 — Supplementary Material 1 [file 13075_2024_3289_MOESM1_ESM.docx]

**Supplementary Material**


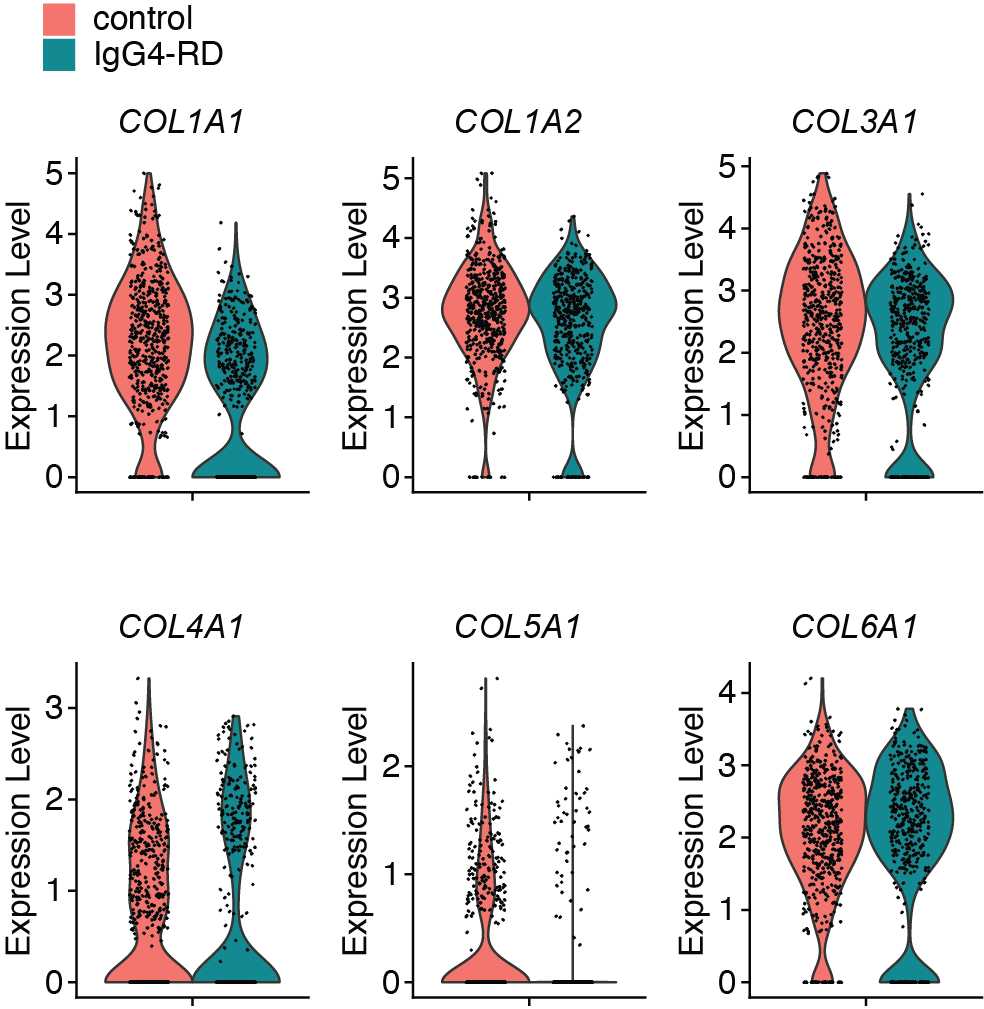


**Supplementary Figure 1.** **Collagen gene expression in the fibroblast cluster.**

The expression of *COL1A1, COL1A2, COL3A1, COL4A1, COL5A1* and *COL6A1* are illustrated by violin plots.


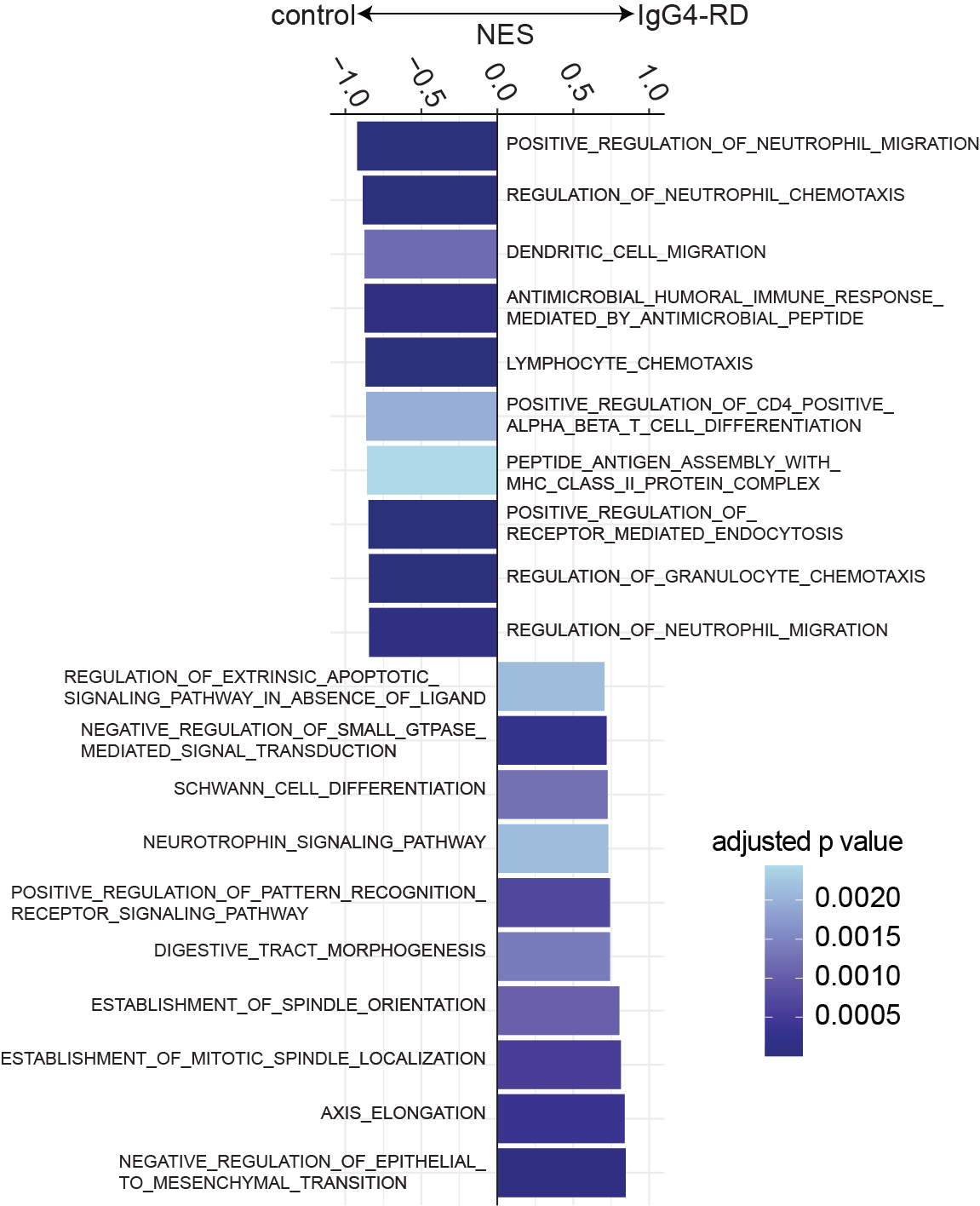


**Supplementary Figure 2. Gene set enrichment analysis of *COL15A1*-positive fibroblasts.**

The bar graph illustrates the enriched KEGG pathways of *COL15A1*-positive fibroblasts.

**Supplementary Table S1. Clinical manifestation of IgG4-RD patients and control subject.**

|  | sex | age (years) | *IgG4 (mg/dL) | **IgG (mg/dL) | affected organs | treatment | assays |
| --- | --- | --- | --- | --- | --- | --- | --- |
| patient 1 | female | 72 | 1670 | 2531 | salivary and lacrimal gland | No Tx | scRNA-seq, IHC |
| patient 2 | male | 80 | 1790 | 4638 | salivary and lacrimal gland | No Tx | scRNA-seq, IHC |
| **^†^**patient 3 | male | 56 | 712 | 2039 | salivary gland, kidney | PSL | IHC, serum |
| **^†^**patient 4 | male | 55 | 996 | 2507 | salivary gland | PSL | IHC, serum |
| **^†^**patient 5 | male | 67 | 1150 | 3631 | salivary and lacrimal gland, sinus | PSL | serum |
| **^†^**patient 6 | male | 65 | 269 | 1288 | salivary and lacrimal gland | RTX | serum |
| **^†^**patient 7 | male | 70 | 140 | 1496 | aorta | PSL | serum |
| **^†^**patient 8 | male | 50 | 346 | 2643 | lung, pharynx | PSL | serum |
| **^†^**patient 9 | male | 74 | 1470 | 3648 | aorta | PSL | serum |
| **^†^**patient 10 | female | 93 | 3190 | 5081 | lacrimal gland, lung | PSL | serum |
| **^†^**patient 11 | male | 64 | 1270 | 2429 | lacrimal gland, sinus | RTX | serum |
| **^†^**patient 12 | female | 65 | 1150 | 3345 | lacrimal gland | PSL | serum |
| **^††^**patient 13 | male | 53 | 397 | 1923 | lacrimal gland | No Tx | serum |
| **^††^**patient 14 | male | 69 | 483 | 1333 | salivary and lacrimal gland | No Tx | serum |
| **^††^**patient 15 | male | 79 | 480 | 2095 | lymph node | No Tx | serum |
| **^††^**patient 16 | male | 69 | 220 | 998 | salivary gland, orbit | No Tx | serum |
| **^††^**patient 17 | male | 66 | 204 | 1225 | salivary and lacrimal gland, lung, bile duct | No Tx | serum |
| control 1 | male | 60 | N.D. | N.D. | (Oropharyngeal cancer) | N.A. | scRNA-seq, IHC |
| control 2 | male | 62 | N.D. | N.D. | (Oropharyngeal cancer) | N.A. | scRNA-seq, IHC |
| control 3 | male | 55 | N.D. | N.D. | (Non-tumor regions of benign salivary gland tumor) | N.A. | IHC |
| control 4 | female | 74 | N.D. | N.D. | N.D. | N.A. | serum |
| control 5 | female | 73 | N.D. | N.D. | N.D. | N.A. | serum |
| control 6 | male | 59 | N.D. | N.D. | N.D. | N.A. | serum |
| control 7 | male | 69 | N.D. | N.D. | N.D. | N.A. | serum |
| control 8 | male | 49 | N.D. | N.D. | N.D. | N.A. | serum |

N.D.: not determined, PSL: prednisolone, RTX: rituximab, Tx, treatment, N.A.: not applicable, scRNA-seq: single-cell RNA sequencing, IHC: immunohistochemistry.

*Serum IgG4 normal range: 11 – 121 mg/dL

**Serum IgG normal range: 870 – 1700 mg/dL

**^†^**Active patients in Figure 2

**^††^**Inactive patients in Figure 2
